# Genomic prediction and genome-wide association study using combined genotypic data from different genotyping systems: application to apple fruit quality traits

**DOI:** 10.1093/hr/uhae131

**Published:** 2024-07-08

**Authors:** Mai F Minamikawa, Miyuki Kunihisa, Shigeki Moriya, Tokurou Shimizu, Minoru Inamori, Hiroyoshi Iwata

**Affiliations:** Institute for Advanced Academic Research (IAAR), Chiba University, 1-33 Yayoi, Inage, Chiba, Chiba 263-8522, Japan; Laboratory of Biometry and Bioinformatics, Department of Agricultural and Environmental Biology, Graduate School of Agricultural and Life Sciences, The University of Tokyo, 1-1-1 Yayoi, Bunkyo, Tokyo 113-8657, Japan; Institute of Fruit Tree and Tea Science, National Agriculture and Food Research Organization (NARO), 2-1 Fujimoto, Tsukuba, Ibaraki 305-8605, Japan; Institute of Fruit Tree and Tea Science, NARO, 92-24 Shimokuriyagawa Nabeyashiki, Morioka, Iwate 020-0123, Japan; Institute of Fruit Tree and Tea Science, NARO, Okitsu Nakacho, Shimizu, Shizuoka 424-0292, Japan; Laboratory of Biometry and Bioinformatics, Department of Agricultural and Environmental Biology, Graduate School of Agricultural and Life Sciences, The University of Tokyo, 1-1-1 Yayoi, Bunkyo, Tokyo 113-8657, Japan; Laboratory of Biometry and Bioinformatics, Department of Agricultural and Environmental Biology, Graduate School of Agricultural and Life Sciences, The University of Tokyo, 1-1-1 Yayoi, Bunkyo, Tokyo 113-8657, Japan

## Abstract

With advances in next-generation sequencing technologies, various marker genotyping systems have been developed for genomics-based approaches such as genomic selection (GS) and genome-wide association study (GWAS). As new genotyping platforms are developed, data from different genotyping platforms must be combined. However, the potential use of combined data for GS and GWAS has not yet been clarified. In this study, the accuracy of genomic prediction (GP) and the detection power of GWAS increased for most fruit quality traits of apples when using combined data from different genotyping systems, Illumina Infinium single-nucleotide polymorphism array and genotyping by random amplicon sequencing-direct (GRAS-Di) systems. In addition, the GP model, which considered the inbreeding effect, further improved the accuracy of the seven fruit traits. Runs of homozygosity (ROH) islands overlapped with the significantly associated regions detected by the GWAS for several fruit traits. Breeders may have exploited these regions to select promising apples by breeders, increasing homozygosity. These results suggest that combining genotypic data from different genotyping platforms benefits the GS and GWAS of fruit quality traits in apples. Information on inbreeding could be beneficial for improving the accuracy of GS for fruit traits of apples; however, further analysis is required to elucidate the relationship between the fruit traits and inbreeding depression (e.g. decreased vigor).

## Introduction

Genomics-based approaches, such as genomic selection (GS) and genome-wide association studies (GWAS), have paved the way for the efficient breeding of plants, especially perennial fruit trees [1, 2]. The long juvenile period of fruit trees increases the time required for breeding, and their large plant size limits the population size for selecting superior genotypes. GS enables the selection of superior genotypes during the juvenile period based on genomic estimated breeding values (GEBVs) calculated from genomic prediction (GP) and allows the selected genotypes to be evaluated in field trials [1]. GS is more effective than conventional marker-assisted selection (MAS), particularly for traits controlled by many genes [3, 4]. GWAS enables the detection of quantitative trait loci (QTLs) for a target trait without using a biparental segregating population [5]. Such genomics-based approaches require high-density genome-wide markers because every QTL must be in linkage disequilibrium (LD) with at least one single-nucleotide polymorphism (SNP) [5, 6].

Advances in next-generation sequencing (NGS) have enabled the development and detection of many high-quality genome-wide markers [2]. Various marker genotyping systems have been developed using genomics-based approaches. SNP genotyping arrays based on Illumina and Affymetrix platforms have been developed for several species of plants [7, 8]. In apples, three systems of single-SNP genotyping arrays, the Illumina 8K [9] or 20K [10], and the Affymetrix Apple480K [7] arrays, have been developed. NGS-based genotyping-by-sequencing (GBS) [11] and restriction site-associated DNA sequencing (RAD-seq) [12], restriction enzyme-based methods, have also been developed and applied to genomics-based approaches for fruit trees [13, 14]. Recently, genotyping by random amplicon sequencing-direct (GRAS-Di) has been proposed as a novel NGS-based and non-targeted polymerase chain reaction (PCR)-based technology [15], but its use for GS and GWAS in fruit trees has not been reported.

As new genotyping platforms are developed, data from different platforms must be combined. It is difficult to reanalyze populations that have been analyzed using previous genotyping tools, because DNA from the populations discarded during selection process cannot be obtained again. Howard *et al.* [16] investigated the compatibility between Illumina Infinium 20K and Affymetrix Axiom 480 K SNP array data in apples. It concluded that 8,412 compatible SNPs might be available to integrate of the Infinium and Axiom SNP array datasets. However, the integration of SNP data obtained from different SNP genotyping systems (e.g. SNP arrays and NGS-based methods) and the potential use of such integrated data for GS and GWAS have not yet been reported.

**Figure 1:**
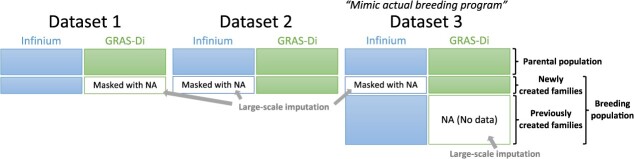
Types of datasets used for genotype imputation and phasing.

**Table 1:** Accuracy of parental phase and genotype score

Method*^a^*	Dataset 1		Dataset 2		Dataset 3	
	Phasing accuracy	Score accuracy*^b^*	Phasing accuracy	Score accuracy*^b^*	Phasing accuracy	Score accuracy*^b^*
Beagle 4.0 combined with an ***improved*** method of estimation for parental phase	**0.80**	**0.92**	**0.86**	**0.99**	**0.86**	**0.99**
Beagle 4.0 combined with a method of estimation for parental phase	0.79	**0.92**	0.85	**0.99**	0.85	**0.99**
Beagle 5.4 combined with an ***improved*** method of estimation for parental phase	0.74	0.82	0.83	0.93	0.83	0.93
Beagle 5.4 combined with a method of estimation for parental phase	0.72	0.82	0.81	0.93	0.80	0.93

a Beagle 4.0 uses pedigree information, but the latest version, Beagle 5.4, does not.

b SNP genotypes were scored as 1, 0, −1 for AA, Aa, aa, respectively.

Apples are one of the most commercially important fruit crops [13]. Most of the apples bred in Japan mainly originate from only seven founders: ‘Ralls Janet’, ‘Delicious’ strains, ‘Golden Delicious’, ‘Jonathan’, ‘Worcester Pearmain’, ‘Indo’, and ‘Cox’s Orange Pippin’ [17]. A few superior founders or leading cultivars (e.g. apple cultivar ‘Fuji’) have been repeatedly used as parents for fruit breeding; therefore, inbreeding coefficients are expected to increase in fruit trees [18]. High inbreeding coefficients increase mortality and decrease vigor in apple [19]. In persimmons, inbreeding depression has been found to occur in yield-related traits, such as fruit weight and tree vigor [20]. The weight of citrus fruit has also been reported to be significantly reduced by inbreeding depression [21]. Xiang *et al*. [22] showed that including the effect of inbreeding depression in the GP model improved the GP accuracy for the total number of piglets born. However, the advantages of GP with inbreeding compared to GP without inbreeding have not been explored for fruit traits.

This study aimed to assess the potential of GS and GWAS for fruit traits using the combined apple datasets from the different genotyping systems, the Infinium SNP array and GRAS-Di systems. Moreover, we aimed to evaluate the performance of the GP considering inbreeding compared to the GP without this effect. Finally, we discussed the genomic regions contributing to inbreeding and fruit quality traits of apples.

## Results

### Phasing and imputation accuracy of combined genotypic data obtained from Infinium and GRAS-Di systems

The Infinium and GRAS-Di marker genotypes (Supplementary Tables S1-S3) were combined based on the physical position of GDDH13. To investigate the influence of the difference between the Infinium and GRAS-Di marker genotype data on the accuracy of genotype phasing and imputation, GRAS-Di (Dataset 1) or Infinium (Dataset 2) marker genotypes of the 15 families were masked as a ‘missing dataset’ (represented as ‘Masked with NA’ in Fig. 1). The phasing and score accuracies for the 15 families in Dataset 2 were slightly higher than those in Dataset 1 (Table 1). The accuracies of the 15 families in Dataset 2 were not different from those in Dataset 3 (Table 1), which mimicked the current state of the actual breeding program. Overall, Beagle 4.0 was more accurate than Beagle 5.4, and the improved estimation method for the parental phase showed superior accuracy compared to our previous method [17]. The combination of Beagle 4.0 and the improved estimation method for the parental phase had the highest phasing (0.86) and score accuracies (0.99) in Datasets 2 and 3.

### GWAS power and GP accuracy when using combined genotypic data

**Figure 2:**
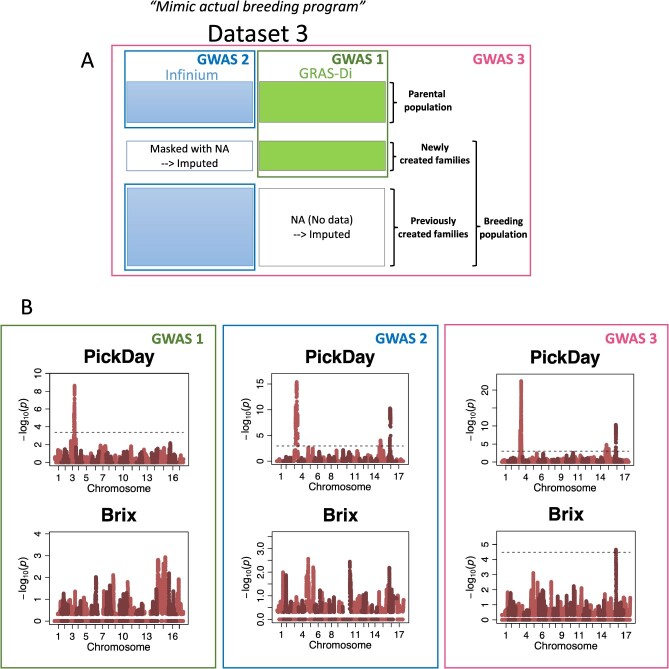
Comparison of GWAS results using three different populations. (A) Three different populations used for GWAS are shown. (B) Manhattan plots for two fruit quality traits. Dashed lines indicate a false discovery rate of 0.05.

**Figure 3:**
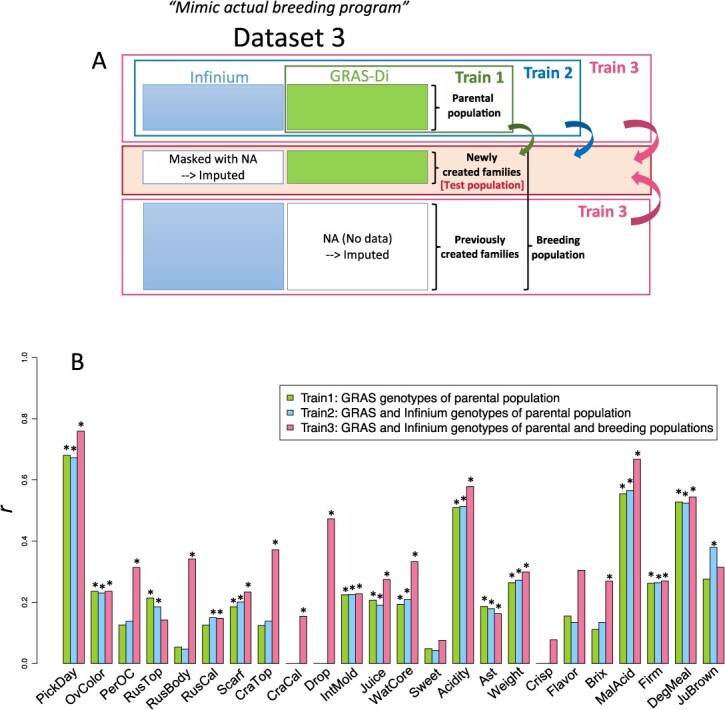
Comparison of genomic prediction using three different training populations. (A) Scheme of genomic prediction using three different training populations. (B) Comparison of prediction accuracy among Train1–3. The prediction accuracy was evaluated using the Pearson’s correlation coefficient (*r*) between the predicted genotypic values and phenotypic values. When the estimated *r* was below zero, it was regarded as zero. Asterisks indicate statistically significant correlations: ^*^p < 0.05.

To assess the potential use of combined genotype data for GWAS and GP, GWAS power and GP accuracy were evaluated using multiple populations (Figs 2A and 3A) for 24 fruit traits (Table 2).

**Table 2:** Fruit quality traits analyzed in this study

Trait	Abbreviation	Description [assesment type]
Harvest time	PickDay	Number of days to harvest from January 1 [17].
Over color	OvColor	Fruit skin redness. Rank: 1 (green/yellow/orange), 2 (pale red), 3 (red/brown), 4 (bright red/purple), 5 (deep red) [visual] [17]
Degree of skin coloration	PerOC	Proportion of fruit skin area colored red. Rank: 1 (<20%), 2 (20% ≤ <40%), 3 (40% ≤ <60%), 4 (60% ≤ <80%), 5 (80%≤) [visual] [17]
Russet top	RusTop	Fruit skin area covered with russet, examined from the top side. Rank: 1 (nil), 2 (little), 3 (mild), 4 (large), 5 (whole) [visual] [17]
Russet body	RusBody	Fruit skin area covered with russet, examined from the horizontal side. Rank: 1 (nil), 2 (little), 3 (mild), 4 (large), 5 (whole) [visual] [17]
Russet calyx	RusCal	Fruit skin area covered with russet, examined from the calyx side. Rank: 1 (nil), 2 (little), 3 (mild), 4 (large), 5 (whole) [visual] [17]
Scarf skin	Scarf	Presence of scarf skin on fruit. 0 (absent), 1 (present) [visual] [17]
Cracking top	CraTop	Presence of fruit cracking, examined from the top side. 0 (absent), 1 (present) [visual] [17]
Cracking calyx	CraCal	Presence of fruit cracking, examined from the calyx side. 0 (absent), 1 (present) [visual] [17]
Preharvest fruit drop	Drop	Degree of fruit drop before harvest time. Rank: 1 (nil), 2 (slight), 3 (moderate), 4 (severe) [visual] [17]
Internal mold	IntMold	Presence of mold in fruit core. 0 (absent), 1 (present) [visual]^17^
Juiciness	Juice	Juiciness during chewing after peeling. Rank: 1 (dry), 2 (slightly dry), 3 (intermediate), 4 (slightly juicy), 5 (juicy) [sensory] [17]
Degree of watercore	WatCore	Degree of watercore observed in equatorial plane of fruit. Rank: 1 (nil), 2 (slight), 3 (moderate), 4 (severe) [visual] [17]
Sweetness	Sweet	Sweetness of peeled fruit. Rank: −2 (weak), −1 (rather weak), 0 (moderate), 1 (rather strong), 2 (strong) [sensory] [17]
Acidity	Acidity	Acidity of peeled fruit. Rank: −2 (weak), −1 (rather weak), 0 (moderate), 1 (rather strong), 2 (strong) [sensory] [17]
Astringency	Ast	Astringency of peeled fruit. 0 (absent), 1 (present) [sensory] [17]
Weight	Weight	Fruit weight (g) [17]
Crispness	Crisp	Crispness of flesh. Rank: 1 (not crisp), 2 (slightly crisp), 3 (rather crisp), 4 (moderately crisp), 5 (crisp), 6 (very crisp), 7 (extremely crisp) [sensory]
Flavor	Flavor	Fruit flavor. Rank: −3 (very bad), 2 (moderately bad), −1 (slightly bad), 0 (neutral), 1 (slightly good), 2 (moderately good), 3 (very good) [sensory]
Soluble solid content	Brix	Brix of the squeezed juice, measured using a refractometer [17].
Malic acid	MalAcid	Acidity of squeezed juice (%), measured as titratable acid content converted into malic acid weight [17].
Firmness	Firm	Mean firmness of sunny and shaded sides of the fruit (Magness-Taylor penetrometer) (lb) [17]
Degree of mealiness	DegMeal	Flesh mealiness after storage for 28–30 days <20°C, measured following the procedure described by Moriya *et al*. [23]
Flesh browning	JuBrown	Browning degree of the fruit juice. Rank: 1 (nil), 2 (slight), 3 (moderate), 4 (strong), 5 (extreme) [visual] [24]

Significant loci were detected for more traits (13 fruit traits analyzed), and the –log_10_(*p*) values of the significant loci were higher in the SNP-set GWAS using Infinium and GRAS-Di markers (GWAS 3; 2,286 individuals in total) than in those using only Infinium markers (GWAS 2; 2095 individuals in total) or GRAS-Di markers (GWAS 1; 353 individuals in total; Fig. 2; Supplementary Figs. S1 to S3; Supplementary Tables S4 to S6). Significant loci on Chr. 15 and Chr. 16 for harvest time (PickDay) were detected in the SNP-set GWAS using Infinium (GWAS 2) or Infinium and GRAS-Di (GWAS 3) markers of the combined populations but were not detected when using the GRAS-Di markers (GWAS 1; Fig. 2; Supplementary Tables S4 to S6). Significant loci on Chr. 16 for soluble solid content (Brix) were detected only in the SNP-set GWAS using Infinium and GRAS-Di markers (GWAS 3). The SNP-set GWAS showed broader peaks in the significant regions than the single-SNP GWAS (Fig. 2B; Supplementary Figs. S3–S5; Supplementary Tables S6 and S7). A significant peak was observed in Chr. 16 for Brix, detected in the SNP-set GWAS, but was not significant in the single-SNP GWAS, although the highest peak was observed for Chr. 16 (Supplementary Figs. S3 and S4). In contrast, a significant peak observed at Chr. 16 for sweetness (Sweet) was detected in the single-SNP GWAS, but not in the SNP-set GWAS. Significant regions in Chr. 16 for Brix overlapped with that for Sweet (phenotypic correlation (*r*) = 0.39; Supplementary Table S8). In the SNP-set GWAS using Infinium markers, a significant peak on Chr. 15 was detected for Sweet (Supplementary Fig. S2).

The GP model trained using the combined Infinium and GRAS-Di markers of the combined parental and breeding populations (Train 3) attained the highest accuracy for 20 of 24 fruit traits compared with that trained using only parental population (Train 1 or Train 2; Fig. 3), whereas the difference in the accuracy between the GP model trained using only GRAS-Di markers (Train 1) and the combined GRAS-Di and Infinium markers (Train 2) of the parental population was small for most of the traits analyzed. The prediction accuracy was high for PickDay (*r* = 0.76) and malic acid (MalAcid; *r* = 0.67) and ﻿intermediate for Acidity (*r* = 0.58) and degree of mealiness (DegMeal; *r* = 0.54).

We also compared the accuracy of the GP model using varying numbers of SNPs (8,929 SNPs within gene regions, 13,122 SNPs within non-gene regions, and the total 22,501 SNPs; Supplementary Fig. S6). The GP model using all 22,501 SNPs demonstrated the highest accuracy for the most traits (8 of 17 traits showing significant (*p* < 0.05) accuracy). Thus, we used the complete SNP set in subsequent studies.

### Inbreeding coefficients in apple population

**Figure 4:**
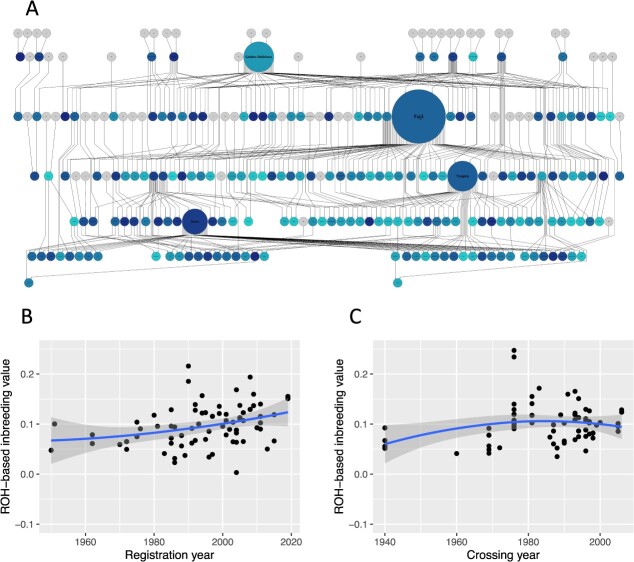
Changes in marker genotype (ROH)-based inbreeding coefficients in apple parental population. (A) Visualization of the marker genotype (ROH)-based inbreeding coefficients on the pedigree of the apple parental varieties. The blue circles indicate the varieties used in this study. Lighter or darker blue circles show higher or lower values, respectively. The size of the circle is based on pedigree contribution. Relation between the marker genotype-based inbreeding coefficients and registration (B) or crossing (C) years of apple parental varieties. Blue lines indicate local polynomial regression fitting with 0.95 confidence intervals indicated in dark gray.

Most of the apples bred in Japan mainly originate from only seven founders, and a small number of superior cultivars (e.g. apple cultivar ‘Fuji’ in Fig. 4A) have been used repeatedly as parents for fruit breeding. Hence, the inbreeding coefficients were expected to increase in the apple population used in this study. Marker genotype (i.e. runs of homozygosity: ROH)- and pedigree-based inbreeding coefficients were visualized in the pedigrees of the apple parental population (Fig. 4A; Supplementary Fig. 7A). ‘Cox’s Orange Pippin’ and ‘Golden Delicious’, both part of the founder cultivars, showed high values of marker genotype-based inbreeding coefficients, but pedigree-based inbreeding coefficients were low in the two cultivars. The marker genotype-based inbreeding coefficients gradually increased with the registration year (Fig. 4B); however, this increase appeared to have been suppressed in recent years with the crossing year (Fig. 4C). This trend was also observed in pedigree-based inbreeding coefficients (Supplementary Figs. 7B and 7C).

### GP considering the effect of inbreeding coefficients

The combined Infinium and GRAS-Di markers of the combined parental and breeding populations were used as a training population (Train 3 in Fig. 3A) to evaluate the potential of GP, considering the inbreeding effect. The GP model that considered the additive and inbreeding effects was superior to the model that considered only the additive effect for over color (OvColor), degree of skin coloration (PerOC), cracking calyx (CraCal), preharvest fruit drop (Drop), internal mold (IntMold), Brix, and DegMeal (higher correlation coefficient (*r*) and lower root-mean-square error (RMSE) values; Table 3). The GP model that considered the additive, dominance, and inbreeding effects also showed higher accuracy than the model without the inbreeding effect. However, no clear improvement in prediction accuracy was observed when the inbreeding effect of the other traits was considered (Supplementary Table S9).

**Table 3:** Prediction accuracy of single- and multi-kernel models

Model	OvColor	PerOC	CraCal	Drop	IntMold	Brix	DegMeal
Additive	0.2362^*^ (0.9093)	0.3139^*^ (1.1960)	0.1544^*^ (0.1480)	0.4727^*^ (0.9948)	0.2276^*^ (0.1222)	0.2692^*^ (1.0358)	0.5441^*^ (0.2147)
Additive and inbreeding	0.2437^*^ (0.8670)	0.3160^*^ (1.1474)	0.1762^*^ (0.1447)	0.4758^*^ (0.9927)	0.2363^*^ (0.1212)	0.3047^*^ (1.0185)	0.5486^*^ (0.2137)
Improvement rate by including inbreeding (Correlation: additive and inbreeding/additive, RMSE: additive/additive and inbreeding)	1.0318 (1.0488)	1.0069 (1.0424)	1.1413 (1.0227)	1.0065 (1.0021)	1.0382 (1.0083)	1.1318 (1.0170)	1.0083 (1.0047)
Additive and dominance	0.2913^*^ (0.7961)	0.3856^*^ (0.9358)	0.1745^*^ (0.1468)	0.3980^*^ (1.0163)	0.2239^*^ (0.1222)	0.2659^*^ (1.0507)	0.5347^*^ (0.2174)
Additive, dominance, and inbreeding	0.2915^*^ (0.7898)	0.3866^*^ (0.9320)	0.1866^*^ (0.1445)	0.4098^*^ (1.0108)	0.2242^*^ (0.1218)	0.2792^*^ (1.0381)	0.5401^*^ (0.2168)
Improvement rate by including inbreeding (Correlation: additive, dominance, and inbreeding/additive and dominance, RMSE: additive and dominance/additive, dominance, and inbreeding)	1.0007 (1.0080)	1.0024 (1.0041)	1.0694 (1.0160)	1.0299 (1.0054)	1.0011 (1.0038)	1.0499 (1.0122)	1.0100 (1.0030)

Prediction accuracy was evaluated using Pearson’s correlation coefficient (*r*) and RMSE (represented in parentheses) for the predicted and observed values. Values in bold signify the greater accuracy (the higher *r* and the lower RMSE values) between the models with and without inbreeding effects for each trait. Asterisks indicate significant correlations: ^*^p < 0.05.

The highest (positive) and lowest (negative) correlation coefficients between the marker-based inbreeding coefficients and phenotypic values were observed for Brix and DegMeal, respectively (Table 4). The phenotypic value of Brix gradually increased with the crossing year, although it remained almost unchanged with the registration year (Supplementary Figs. S8 and S9). In contrast, the phenotypic value of DegMeal gradually decreased over the registration and crossing years, with a slight increase in recent years.

**Table 4:** Correlation between inbreeding coefficient and phenotypes

Fruit trait	Correlation coefficient (*r*)
PickDay	0.21^*^
OvColor	−0.09^*^
PerOC	−0.05^*^
RusTop	0.05^*^
RusBody	0.05^*^
RusCal	−0.02^*^
Scarf	−0.07^*^
CraTop	0.03
CraCal	0.07^*^
Drop	−0.07^*^
IntMold	0.08^*^
Juice	−0.01
WatCore	0.08^*^
Sweet	−0.06^*^
Acidity	−0.06^*^
Ast	0.00
Weight	−0.18^*^
Crisp	0.03
Flavor	−0.03
Brix	**0.31** ^*^
MalAcid	−0.17^*^
Firm	0.22^*^
DegMeal	**−0.24** ^*^
JuBrown	−0.15

Asterisks indicate significant correlations:

^*^ *p* < 0.05. Values in bold signify the highest or lowest correlation coefficients.

The fixed effects of inbreeding in the model that considered additive, dominance, and inbreeding were positive and negative for Brix and DegMeal, respectively (Supplementary Table S10). The positive and negative inbreeding effects translated into a 1.189% increase in Brix and 0.095 decrease in DegMeal for the child of an individual, and a 0.594% increase in Brix and 0.047 decrease in DegMeal for the grandchild of an individual (Supplementary Table S10).

### ROH islands in the apple genome

**Figure 5:**
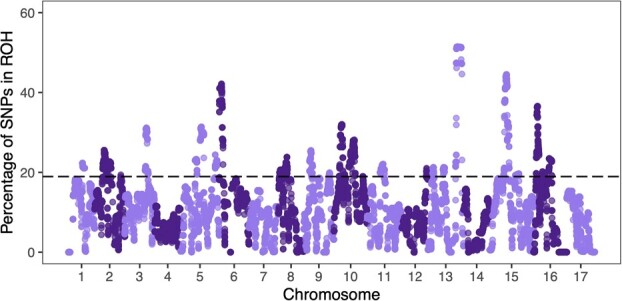
Frequency (percent) of SNP occurrence within ROH regions. The dashed line indicates the adopted threshold (top 15% of the SNP frequencies), which defines ROH islands.

To determine the occurrence of contiguous homozygous segments across the apple genome, ROH analyses were conducted using all the combined datasets. Significant (top 15% of the SNP frequencies) ROH islands were detected in most chromosomes, except for Chr. 4, Chr. 7, Chr. 14, and Chr. 17 (Fig. 5; Supplementary Table S11). The most significant ROH peak with the highest frequency (percent) of occurrence within the ROH regions was located on Chr. 13, followed by Chr. 15, Chr. 6, Chr. 16, and Chr 10. Of these, significant ROH loci were found on Chr. 15 overlapped with the significant SNP loci for Drop detected in the GWAS (Supplementary Fig. S3; Supplementary Table S6). The significant GWAS peaks for Chr. 16 for cracking top (CraTop), juiciness (Juice), degree of watercore (WatCore), Acidity, Brix, MalAcid, and flesh browning (JuBrown) (Supplementary Fig. S3; Supplementary Table S6), and PickDay (Supplementary Fig. S2; Supplementary Table S5), and Sweet (Supplementary Fig. S4; Supplementary Table S7) resided within the ROH islands. In contrast, significant GWAS peaks for Chr. 10 for Drop and DegMeal (Supplementary Fig. S2; Supplementary Table S5), and internal mold (IntMold; Supplementary Fig. S1; Supplementary Table S4) resided within the ROH islands.

## Discussion

In this study, the accuracy of GP and the detection power of GWAS increased when combined genotypic data from different genotyping systems, the Infinium SNP array and GRAS-Di systems were used. This outcome is supported by high-performance phasing and imputation algorithm of Beagle. Beagle 4.0 [25], combined with the improved estimation method for the parent stage, showed the highest phasing (0.86) and score accuracy (0.99) in Dataset 3 among the methods used in this study. The higher phasing accuracy of Beagle 4.0, compared to the findhap.f90 [26], MaCH [27], and fastPHASE [28] programs, was also shown in our previous study using an apple population [17]. The high phasing ability of Beagle 4.0 may be related to its high imputation accuracy. Several studies have reported the Beagle algorithm’s high imputation accuracy. Beagle 3.3.2 has retained a higher (>0.95) imputation accuracy than any of the machine learning methods in simulated and real pig datasets with a high missing rate (0.90) [29]. At the same time, Yang *et al*. [30], using simulation data, reported that the imputation accuracies of Beagle (version was not written) were 0.95 and 0.76 for missing rates of 0.60 and 0.80, respectively. In this study, Beagle 5.4 was less accurate than Beagle 4.0, although Beagle 5.4 was the latest version; this may be because Beagle 4.0 uses pedigree information, whereas the new version. 5.4 does not. The importance of using pedigree information has been demonstrated to result in higher imputation accuracy on dairy cattle [31].

The combination of Infinium and GRAS-Di data enabled an increase in the number of samples and markers, which may also have contributed to the improvement in the accuracy of GP and the detection power of GWAS. In general, the accuracy of GP and the detection power of GWAS increase when the sample size or marker density is large [32–34]. The present study’s GP model trained using the GRAS-Di and Infinium markers (22,051 SNPs) of the combined parental and breeding populations (2,216 individuals) attained the highest accuracy for most fruit traits. However, the difference between the accuracy of the GP model trained using only GRAS-Di markers (10,899 SNPs) and that trained using the combined GRAS-Di and Infinium markers (22,051 SNPs) in the parental populations (184 individuals) was small; this implies that population size could be more important than marker density in improving the accuracy of GP. Jung *et al*. [35] reported that a study using European apples achieved predictive ability at 10,000 SNPs for harvest date and floral emergence. Increasing the sample size may also have contributed to the higher detection power of the GWAS using combined populations in this study. More significant loci were detected in the GWAS using >2,000 individuals than in the GWAS using 353 individuals. A larger dataset with tens of thousands of individuals is required to detect more causal variants with small genetic effects, as reported for humans [36]. Wood *et al*. [36] identified 697 significant variants that explained one-fifth of the heritability of adult height using a GWAS of 253,288 individuals.

The SNP-set and single-SNP GWASs detected several significant associations for the 12 common fruit traits. However, significant associations for Brix and Sweet were detected only in the SNP-set and single-SNP GWASs, respectively; this implies that both methods are useful for detecting QTLs of these traits. Hamblin and Jannink [37] suggested that SNP-set and single-SNP GWASs are indispensable for detecting QTLs for traits with low-to-moderate heritability, as QTL may be detected with one method but not the other.

The SNP-set GWAS using Infinium and GRAS-Di markers of the combined parental and breeding populations detected significant associations for fruit traits. A significant association on Chr. 16 for Brix was consistent with the associations of CraTop, Juice, Acidity, MalAcid, and JuBrown. This significant region also overlapped with that of Sweet, which was detected in a single-SNP GWAS. The results suggested a close genetic linkage between the QTLs controlling the traits, resulting in moderate to high phenotypic correlations between the traits (*r* = 0.39 between Brix and Sweet; *r* = 0.81 between MalAcid and Acidity). Significant SNPs can be used as common markers for these traits in MAS programs [38].

Sweetness and acidity play important roles in determining fruit taste; therefore, they are important qualities for apple breeders [39]. Due to the complexity of evaluating sweetness and acidity, soluble solid content (SSC) expressed as Brix and titratable acid content (TA) are commonly used to estimate these traits. Several candidate genes for TA have been reported; however, only a few candidate genes for SSC have been identified until recently [40]. For TA, two major QTLs, *Ma* (*malic acid*; Chr 16) and *Ma3* (Chr 8), have been identified in apples, and both loci jointly explain 66% of the total variation in TA [41]. Malic acid is the predominant organic acid, accounting for up to 90% of the total organic acids [40]. A strong candidate gene *Ma1* in the *Ma* locus of Chr 16 encodes a protein closely related to aluminum-activated malate transporter 9 (ALMT9) in *Arabidopsis thaliana*, and the truncation of *Ma1* is associated with low TA in apple [42–44]. Several candidate genes have also been proposed for the *Ma3* locus of Chr 8, such as *MdMYB4* and *MdME* by Sun *et al*. [45], and *MdSAUR37*, *MdPP2CH*, and *MdALMTII* by Jia *et al*. [46]. These QTLs overlapped with the significant associations in Chr. 8 and Chr. 16 for Acidity and MalAcid, respectively; this result suggests that the candidate genes for TA identified in a previous study could also be useful for improving Acidity. The high correlations between acidity and TA (*r* = 0.81 in this study) have also been reported by Harker *et al*. [47] (*r* = 0.86) and Kouassi *et al*. [48] (0.75 ≤ *r* ≤ 0.78).

For SSC, the major LGs have been identified as LGs 1 and 3 in apple [40, 49]. Recently, several candidate SSC genes have been identified. The SNP variation in the promoter region of the gene *MdSDH2* on LG 1 affects its binding to the transcription factor MdABI3, which leads to the downregulation of the expression level of *MdSDH2* and finally reduces the fructose content [50]. In addition, SNP variations in the gene *MdSOT2* on Chr 10 dramatically reduced SSC in apple fruit [51]. In contrast to TA, the weak correlations (0.23 ≤ *r* ≤ 0.41) between sweetness and SSC have been reported in previous studies [47, 48]. This study found a moderate correlation between Sweet and Brix (*r* = 0.39). Therefore, these QTLs/genes may not improve sweetness, but may improve SSC. However, the common significant SNPs on Chr 16 for Sweet and Brix, detected in the GWAS in this study, could be used to improve both traits. For Sweet, significant SNPs were also identified on Chr 15, suggesting that the QTL could also be a candidate for the improvement of Sweet. Finding an appropriate level of sweetness and acidity and a proper balance between the two is essential for successful apple breeding because the sweetness is influenced by acidity, just as apples with low acidity are perceived as sweet. In contrast, apples with high acidity are perceived as less sweet.

The inbreeding coefficients increased in the apple parental population as the registration or breeding year progressed; this may be because most of the apple varieties bred in Japan originated from only seven founders, and a small number of superior cultivars have been used repeatedly as parents for fruit breeding [18]. Inbreeding coefficients gradually increased over the crossing years. However, this increase appears to have been suppressed recently; this implies that the apple breeders could choose to cross parents that do not increase the inbreeding coefficient because higher inbreeding coefficients cause increased mortality and decreased vigor in apples [19]. In addition, this may be related to the fact that from the mid-1990s, apple breeders began again to use introduced varieties with different pedigrees from Japanese varieties (e.g. ‘Coop29’, ‘Scarlett O’Hara’, ‘8H-2-26’, ‘Silken’, and apple genetic resource (JP114069); Supplementary Table S1). The inbreeding coefficients of ‘Cox’s Orange Pippin’ and ‘Golden Delicious’, both part of the founder cultivars, were high in marker genotype-based calculations but low in pedigree-based calculations. The pedigree-based calculations using only the apple varieties used in Japanese breeding programs do not consider inbreeding from their ancestors. However, they might have inherited some degree of inbreeding from its ancestors. In the pedigree-based calculations, the inbreeding of the founders is lower than actual inbreeding; therefore, the inbreeding of the founder’s progenies would also be lower than marker genotype-based calculations [52].

Combining genotyping data from different genotyping systems increases the number of individuals required for training the GP model, which increases the accuracy of the GP model. Considering inbreeding effects, the GP model further improved the accuracy of seven fruit traits (OvColor, PerOC, CraCal, Drop, IntMold, Brix, and DegMeal). Improvement of the GP accuracy by including the inbreeding effect in the GP model has also been reported in pig [22].

The most significant ROH peak was detected in Chr. 13, followed by Chr. 15, Chr. 6, Chr 16, and Chr 10. The most significant ROH island in Chr. 13 (Chr 13:36,473,014– 37,179,149), previously reported QTLs have not been found, although several candidate QTL/genes have been reported near the ROH island in apples (Chr 13: 6,049,060 for crispness and juiciness [53]; Chr 13:1,889,560 for ripening period [54]; Chr 13: 19,381,784– 19,382,359 (i.e. *MdSUP* gene; gene id: MD13G1209600) for linoleic acid in fruit pulp [55]). In contrast, the significant ROH loci on Chr. 15 overlapped with the significant SNP loci for Drop detected by the GWAS. In addition, the ROH islands on Chr. 10 and Chr. 16 overlapped with the significantly associated regions for nine and three fruit traits, respectively. These results suggest that the significant regions on the chromosomes may have been exploited by breeders for the selection of promising apples, resulting in increased homozygosity in these regions [56]. These regions of homozygosity could be related to inbreeding depression (e.g. decreased vigor [19]), which may be related to the high correlation between inbreeding and Brix or DegMeal. However, further analysis is required to elucidate the relationship between the fruit traits and inbreeding depression (e.g. decreased vigor [19]), because these fruit traits are unlikely to be directly related to inbreeding.

In conclusion, we demonstrated the potential of GS and GWAS for fruit traits using the combined apple datasets from the different genotyping systems. The accuracy of GP and the detection power of GWAS increased when combined genotypic data were used. In addition, the GP model, which considers the effect of inbreeding, further improved the accuracy of the seven fruit traits. Using the combined data from the multiple genotyping systems and considering inbreeding could demonstrate Darwin’s statement, ‘Our oldest cultivated plants, such as wheat, still often yield new varieties: our oldest domesticated animals are still capable of rapid improvement or modification.’ [57, 58]. GS with high accuracy can identify superior genotypes from numerous individuals during the seedling stage. In addition, precise GWAS can detect QTLs for a target trait and can facilitate the development of DNA markers for conventional MAS. This would address challenges such as large plant size and long juvenile period in fruit trees and greatly contribute to promote the efficiency of fruit tree breeding.

## Materials and methods

### Plant materials

A total of 184 apple cultivars and breeding lines of apple (*Malus* × *domestica* Borkh.), called the parental population (Supplementary Table S1), of which 22 lines were only used for genotyping using the Infinium [10] and GRAS-Di [15] methods because of missing phenotypes. We also used 207 full-sib families consisting of 2,223 F_1_ individuals, called breeding populations (Supplementary Table S2). Of the 207 families, 15 (191 F_1_ individuals) were genotyped using the Infinium and GRAS-Di methods, whereas the other 192 families (2,032 F_1_ individuals) were genotyped using only the Infinium method. Some lines in the breeding population were only used for genotyping using the Infinium and/or GRAS-Di methods because of missing phenotypes (Supplementary Table S2). The breeding populations were derived from crosses between the 61 parental cultivars and breeding lines included in the parental population. In each of the 76 families, there were ≥10 individuals per family; however, the remaining families had <10 individuals because of the elimination of several individuals during the breeding process. These plant materials were grafted onto popular rootstocks (JM1, JM7, M26, etc.) and cultivated for at least 4 years for phenotypic evaluation. All materials mentioned above were cultivated in the experimental orchard of the Institute of Fruit Tree and Tea Science, NARO, in Morioka, Japan.

### Fruit assessment

Detailed information on the phenotypic evaluation of the 24 traits is presented in Table 2. The traits of the parental population were evaluated from 1990 to 2019: some varieties were evaluated for 30 years, while some were evaluated for only 1 year (the average was 7.7 years per cultivar). The breeding population was also evaluated from 2000 to 2019, except for 2009. Almost all of the progenies were evaluated in only 1 year, except 40 F_1_s of ‘Orin’ × ‘Akane’, which were observed for three consecutive years. Two to five fruits, on average, per variety were used for the evaluation. Individual fruits were evaluated separately for weight, firmness (Firm), and degree of mealiness (DegMeal), and the average values were used as phenotypic data. Other traits were evaluated using representative scores/values of the pooled samples or juice. The data for each variety was adjusted considering the effect of the year using the program ‘allEffects’ of R package ‘effects’ as described in Moriya *et al*. [59].

### SNP genotyping using GRAS-Di and Infinium systems

Genomic DNA from the parental and breeding populations was obtained from the leaves using a Genomic-tip 20/G kit and DNeasy Plant Mini Kit (Qiagen, Hilden, Germany), or the CTAB method was implemented using the automated device PI-50α (Kurabo, Osaka, Japan).

According to the manufacturer’s instructions, the genotypes of 18,019 SNPs were determined for all samples, as reported by Bianco *et al*. [10], using the Infinium assay kit (Illumina, San Diego, CA, USA). SNPs that showed unclear cluster separation or no polymorphisms among the tested samples were removed from the analysis by visual inspection. Finally, 11,152 SNPs were obtained from the parental and breeding populations using the Infinium system (Supplementary Tables S1 to S3). The loci of the Infinium SNPs were estimated by the alignment of the SNP probe sequences to the reference genomes, ‘GD’ doubled-haploid line (GDDH13) v1.1 [60]. The rate of missing SNP genotypes was 0.005.

SNP genotypes of the parental population and 15 families (191 F_1_ individuals) of the breeding population were obtained using genotyping by random amplicon sequencing, direct (GRAS-Di) system. This technology was developed by Enoki and Takeuchi [15], and its Patent ID is P2018-42548A. This technology consists of genome-wide target amplification using high-concentration random primers, NGS, and data analysis. An NGS library was constructed using two sequential PCR steps. The first PCR was performed with 63 primers, including Illumina Nextera adaptor sequences and three-base random oligomers, followed by second PCR with indexing primers, including the Illumina multiplexing dual index and P7/P5 adapter sequence. PCR and purification were carried out following the method described by Hosoya *et al*. [61], and the constructed library was sequenced on NovaSeq6000 using the NovaSeq6000 S4 Reagent Kit (paired-end, 150 bp). Trimming was carried out using GRAS-Di software (TOYOTA, Aichi, Japan), with default settings: Nextera adaptor and other Illumina primer sequences were clipped, low-quality reads were removed, and 10 bp at 5′ end of reads were trimmed. Library preparation, sequencing, and data trimming were performed by Eurofins Genomics, Inc. (Tokyo, Japan).

Trimmed reads were mapped onto the apple reference genome GDDH13 v1.1 [60] using BWA-mem of BWA v 0.7.17 with parameters *–t1, –T30, –M, –R* [62]. Subsequently, properly mapped read pairs with high mapping quality (Q > 30) were selected using samtools v 1.11 [63]. From combined data, variants were detected and filtered with the following options, –*QD < 2.0*, *–FS > 60.0*, *–MQ < 30.0*, *–MQRankSum < −12.5*, *–ReadPosRankSum < −8.0*, using GATK-4.1.3.0 [64]. Low-quality variants were excluded using vcftools-0.1.13 [65] based on the following criteria: genotyped in <95% of individuals, read quality <20, minor allele frequency <0.05, and read depth <8.

The overlapped nine markers between GRAS-Di and Infinium systems (mean genotype consistency was 0.98) were removed because the nine GRAS-Di markers exhibited a higher missing rate (0.016) compared to the Infinium markers (0.000). Finally, 10,899 SNPs were obtained from the parental and breeding populations using the GRAS-Di system (Supplementary Tables S1 to S3). The rate of missing SNP genotypes was 0.016.

### Combination of genotypic data obtained from the Infinium and GRAS-Di systems

Because the positional information for the Infinium and GRAS-Di marker genotypes was obtained from the same reference genome, GDDH13, the genotypic data of the two markers were combined based on the positional information. We prepared three different datasets for subsequent analyses. The first (Dataset 1) and second (Dataset 2) datasets consisted of the parental population and 15 families from the breeding population, which were genotyped using both the Infinium and GRAS-Di systems (Fig. 1; 375 individuals in total). To investigate the influence of the difference between the Infinium and GRAS-Di marker genotypic data on the accuracy of genotype phasing and imputation, GRAS-Di (Dataset 1) or Infinium (Dataset 2) marker genotypes of the 15 families were masked as a ‘missing dataset’ (represented as ‘NA’ in Fig. 1). Dataset 3 mimicked the current state of the actual breeding program, in which marker genotypic data obtained from different genotyping systems may exist (Fig. 1; 2,407 individuals in total). In Dataset 3, we assumed breeders were considering switching from the conventional Infinium array system to the new NGS-based GRAS-Di genotyping system. The parental population was genotyped using the Infinium and GRAS-Di systems, whereas the breeding population was assumed to be genotyped using only one of the methods. The GRAS marker genotypes of the 15 families of the breeding population were assumed to be newly created in the breeding program. The Infinium marker genotypes of the families were masked as a ‘missing dataset’. The remaining families in the breeding population had only Infinium marker genotypes and were assumed to have been previously created in the breeding program.

The masked marker genotypes and the sporadically missing genotypes in Datasets 1–3 were imputed and phased using Beagle ver. 4.0 [25] and ver. 5.4 [66, 67]. Beagle 4.0 uses pedigree information; however, the new version 5.4 does not. The parental phases of the individuals were estimated as described by Minamikawa *et al*. [17]. A slightly modified program for estimating the parental phases was also evaluated. In the modified program, a range of 30 bp was randomly selected in one of the two phases, and correlations between the range and those of female and male parents were calculated; then this procedure was repeated 100 times. Finally, the phase is estimated to be inherited from the female (or male) parent with the higher number of times that the correlation was high.

To evaluate the accuracy of genotypic imputation and phasing, the 15 imputed and phased families in each dataset were compared with the original dataset, where the sporadic missing genotypes were imputed and phased (i.e. the imputed and phased GRAS-Di data of 15 families of Dataset 1 were compared with those of Dataset 2, whereas the imputed and phased Infinium data of 15 families of Datasets 2 and 3 were compared with those of Dataset 1).

### GWAS

We used two linear mixed model (LMM)-based GWAS methods, single-SNP GWAS and SNP-set GWAS, implemented in the functions ‘RGWAS.normal’ and ‘RGWAS.multisnp’ of the R package RAINBOWR ver. 0.1.29 [68]. SNP-set GWAS has been reported to outperform single-SNP GWAS because it controls false positives better than the single-SNP method. To avoid spurious associations due to population structure and family relatedness, a kinship matrix and the scores of principal components (PCs) were included in both GWAS models. The kinship matrix was computed in the ‘calcGRM’ functions of RAINBOWR. The number of PCs and the minor allele frequency (MAF) values were set to 3 and 0.025, respectively, for both models. In the SNP-set method, 41 or 101 SNPs were regarded as a single SNP-set with a sliding window (the hyperparameters were set to $window. size. half=20$ and $window. slide=1$). The other hyperparameter values were default values. Significant SNPs were determined using the threshold of false discovery rate (FDR) < 0.05.

The power of GWAS was compared among the three datasets (Fig. 2): (1) The parental population and 15 families of the breeding population, which was genotyped with GRAS-Di system only (GWAS 1); (2) The parental population and 192 families (except the 15 families) of the breeding population, which was genotyped with Infinium system only (GWAS 2); (3) The parental population and all families of the breeding populations, where genotypic data obtained from the Infinium and GRAS-Di systems were combined (GWAS 3).

### Calculation of inbreeding coefficients

Pedigree- and marker genotype (i.e. ROH)-based inbreeding coefficients for the apple parental and breeding populations were computed using the functions ‘pedInbreeding’ and ‘segInbreeding’ of R package optiSel ver. 2.0.5 [69]. Changes in the inbreeding coefficients in the parental population were visualized using the Helium software. The inbreeding coefficients were plotted against the registration or crossing years of the apple cultivars and breeding lines (Supplementary Table S1). Local polynomial regression was conducted using the ‘geom_smooth’ function of the R package ggplot2 ver 3.3.5 [70] to model the relationship between the inbreeding coefficients and registration or crossing years.

### Genomic prediction

A single-kernel model that considers an additive effect was used to compare the accuracy of genomic prediction for the 15 pedigrees of the breeding population using three different training populations (Fig. 3): (1) The parental population, which was genotyped with GRAS-Di system only (Train 1); (2) The parental population, which was genotyped with Infinium and GRAS-Di systems (Train 2); (3) The parental population and all families of the breeding populations, where genotypic data obtained from the Infinium and GRAS-Di systems were combined (Train 3). In addition, we compared the single-kernel models using varying numbers of SNPs; 8,929 SNPs within gene regions, 13,122 SNPs within non-gene regions, and the total 22,501 SNPs. Gene location was obtained from the Genome Database for Rosaceae (GDR) (https://www.rosaceae.org) [71]. The single-kernel model was implemented in the R package BGLR ver. 1.0.9. The additive relationship matrix (kinship matrix) was computed by the ‘A.mat’ function of the R package rrBLUP ver. 4.6.1 [72].

To consider the effect of inbreeding depression (i.e. directional dominance), a single-kernel LMM that considers not only the additive effect (treated as a random effect) but also the effect of inbreeding (treated as a fixed effect) [22] was used for comparison with the single-kernel model that considers only the additive effect. We also evaluated the accuracy of the GP with and without considering the effect of inbreeding (treated as a fixed effect) in a multi-kernel LMM that considered both additive and dominant effects (each treated as a random effect) [22]. The fixed effect of inbreeding (*b*; represented as ‘inbreeding depression parameter’ in Xiang *et al*. [22]) for each trait, which was estimated from the multi-kernel LMM that considers additive, dominance, and inbreeding depression, was listed in Supplementary Table S10. Marker genotype-based inbreeding coefficients were used in the GP model. The dominance relationship matrix was calculated as described by Minamikawa *et al*. [73]. The accuracies of the models were evaluated using a prediction method (Train 3), as shown in Fig. 3. Prediction accuracy was defined using the Pearson’s correlation coefficients (*r*) and RMSE between the observed and predicted values. When the estimated *r* was less than zero, it was regarded as zero.

### Detection of runs of homozygosity islands

The ROH analysis was performed using combined parental and breeding populations. The function ‘consecutiveRUNS.run’ of the R package detectRUNS ver. 0.9.6 [74] was used for analysis. The consecutive-SNP-based approach has been preferred to the sliding-window-based approach to avoid introducing artificial ROHs that are shorter than the window [75]. The parameters of the consecutive-SNP-based method were set as min SNP = 100, maxGap = 10^6^, and minLengthBps = 10^6^, maxOppRun = 1, and maxMissRun = 1. The top 15% of the most frequent SNPs were selected to define the possible ROH islands in the genome.

## Acknowledgements

We are grateful to all the members of the NARO Institute of Fruit Tree and Tea Science for maintaining the apple trees. This research was supported by a grant from the Ministry of Agriculture, Forestry and Fisheries of Japan (Genomics-based Technology for Agricultural Improvement, NGB-2007 and 2010), Cabinet Office, Government of Japan, Cross-ministerial Strategic Innovation Promotion Program (SIP), ‘Technologies for Smart Bio-industry and Agriculture’ (funding agency: Bio-oriented Technology Research Advancement Institution, NARO), MAFF commissioned project study on ‘Smart breeding technologies to Accelerate the development of new varieties toward achieving “Strategy for Sustainable Food Systems, MIDORI”’ Grant Number JPJ012037 and a Grant-in-Aid for JSPS Research Fellow (JP22K20577 and JP23K13928).

## Author contributions

M.F.M. and M.K. conceived and designed the study. M.K. and S.M. extracted DNA and performed SNP genotyping. T.S. helped with SNP genotyping. S.M. performed the phenotyping. M.I. improved the estimation algorithm for the parental phase. M.F.M. combined the genotypes obtained using different genotyping systems and performed GP and GWAS. H.I. provided technical help for the statistical analysis. M.F.M., M.K., and S.M. drafted the manuscript. All the authors have read and approved the manuscript.

## Data availability

Information on DNA markers is available from Supplementary Data Tables.

## Conflict of interest statement

None declared.

## Competing financial interests

The authors declare no competing financial interests.

## Supplementary Data

Supplementary data is available at *Horticulture Research* online.

## Supplementary Material

Web_Material_uhae131
