# Supplementary material for: Genomic prediction and genome-wide association study using combined genotypic data from different genotyping systems: application to apple fruit quality traits
Source: Hortic Res. 2024 Jul 8;11(7):uhae131. doi: 10.1093/hr/uhae131 (PMC11228094; doi:10.1093/hr/uhae131)
Supplement: Web_Material_uhae131 [file web_material_uhae131.zip › 240327_Apple_SupplementaryFigures_Revised.pdf]

Supplementary information

Genomic prediction and genome-wide association study using combined genotypic data from different genotyping systems: Application to apple fruit quality traits.

Mai F. Minamikawa[1,2*], Miyuki Kunihisa[3], Shigeki Moriya[4], Tokurou Shimizu[5], Minoru Inamori[2], Hiroyoshi Iwata[2]

[1]Institute for Advanced Academic Research (IAAR), Chiba University, 1-33 Yayoi, Inage, Chiba, Chiba 263-8522, Japan
[2]Laboratory of Biometry and Bioinformatics, Department of Agricultural and Environmental Biology, Graduate School of Agricultural and Life Sciences, The University of Tokyo, 1-1-1 Yayoi, Bunkyo, Tokyo 113-8657, Japan
[3]Institute of Fruit Tree and Tea Science, National Agriculture and Food Research Organization (NARO), 2-1 Fujimoto, Tsukuba, Ibaraki 305-8605, Japan
[4]Institute of Fruit Tree and Tea Science, NARO, 92-24 Shimokuriyagawa Nabeyashiki, Morioka, Iwate 020-0123, Japan
[5]Institute of Fruit Tree and Tea Science, NARO, Okitsu Nakacho, Shimizu, Shizuoka 424-0292, Japan

*To whom correspondence should be addressed:
Email: minamikawa@chiba-u.jp

Supplementary Figures S1-S9

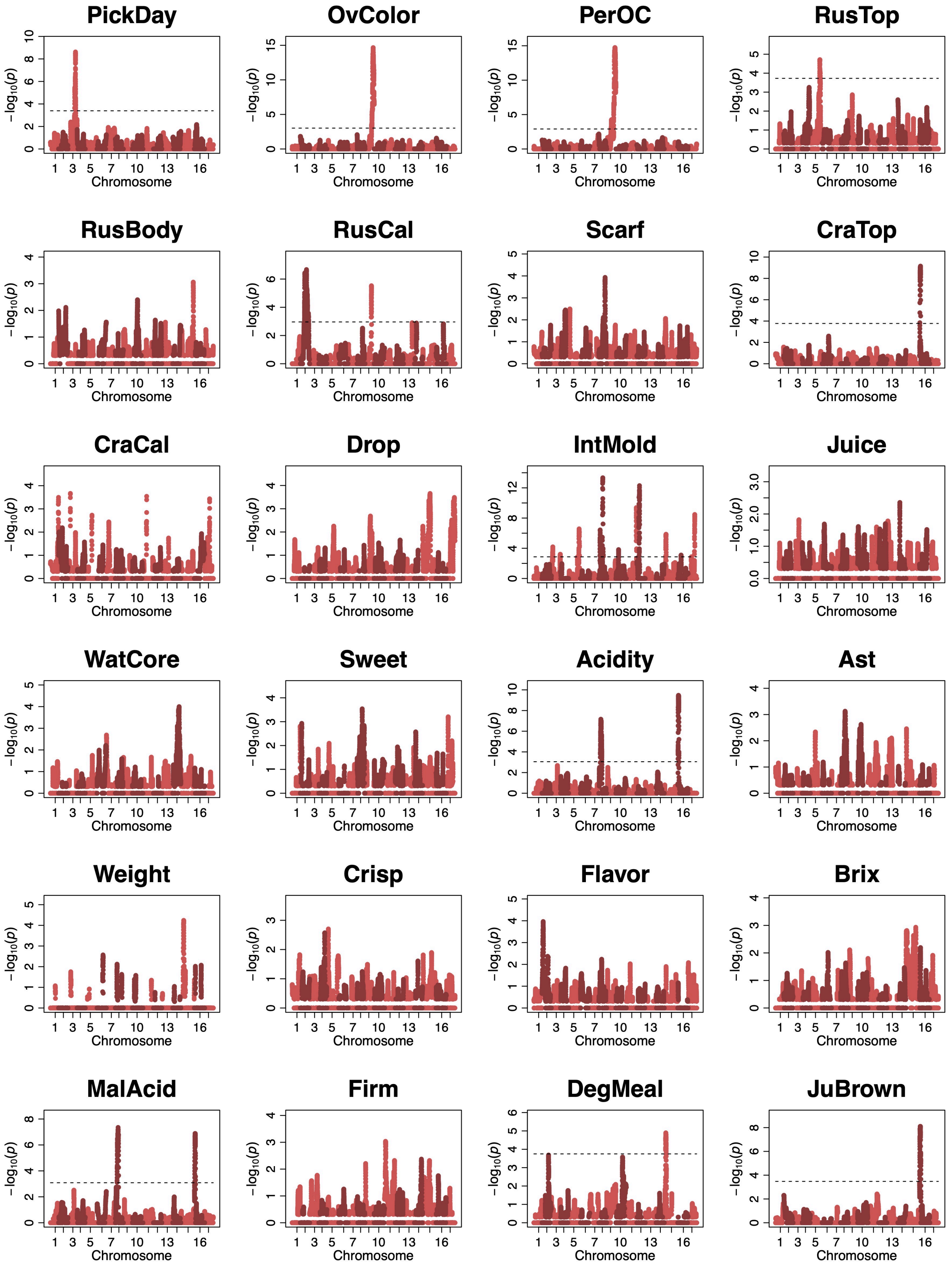

Supplementary Figure S1. SNP-set GWAS using GRAS-Di marker dataset. The GRAS-Di marker set data is shown in Fig. 2A. Dashed lines indicate a false discovery rate of 0.05.

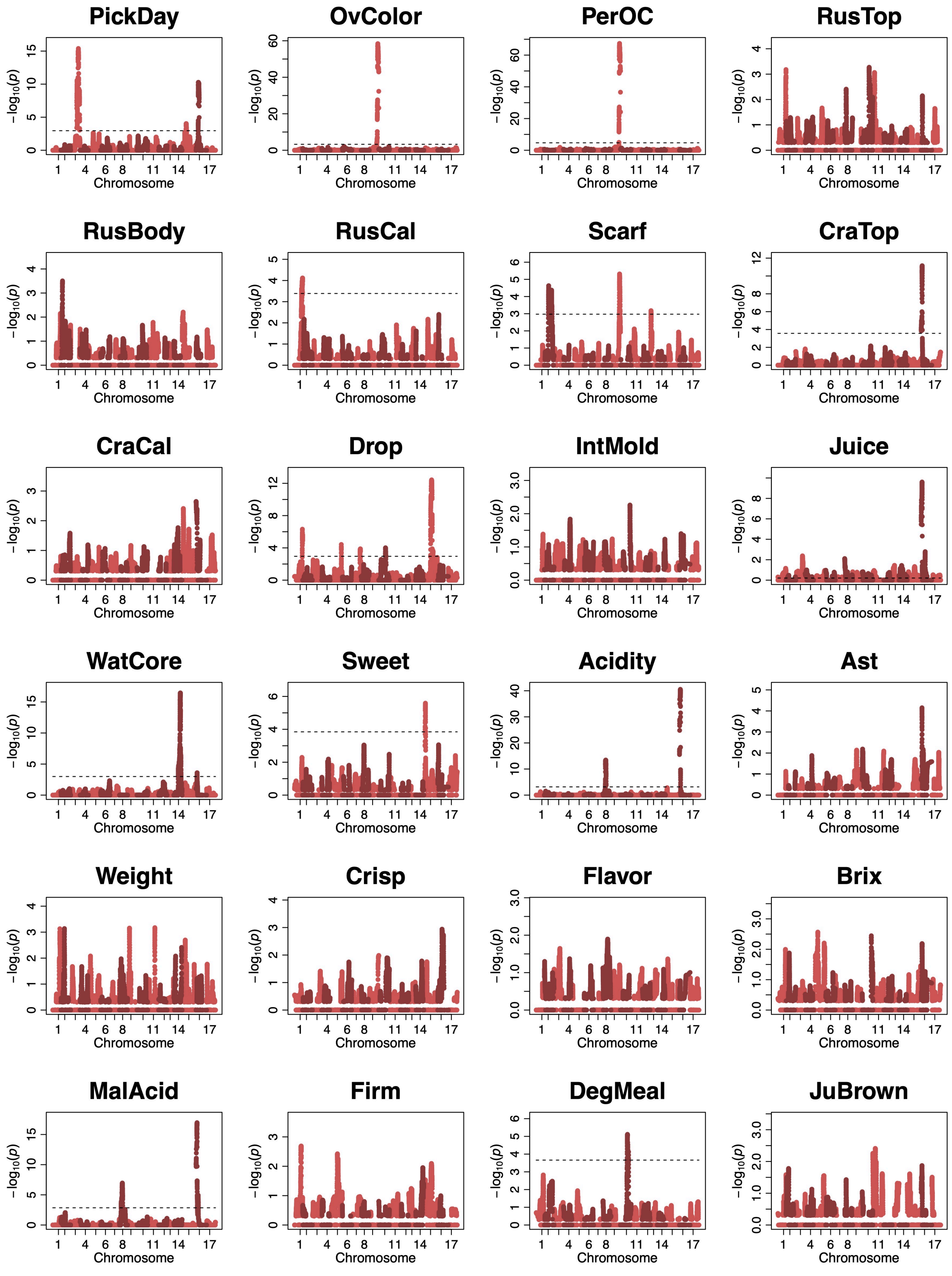

Supplementary Figure S2. SNP-set GWAS using Infinium marker dataset. The Infinium marker set data is shown in Fig. 2A. Dashed lines indicate a false discovery rate of 0.05.

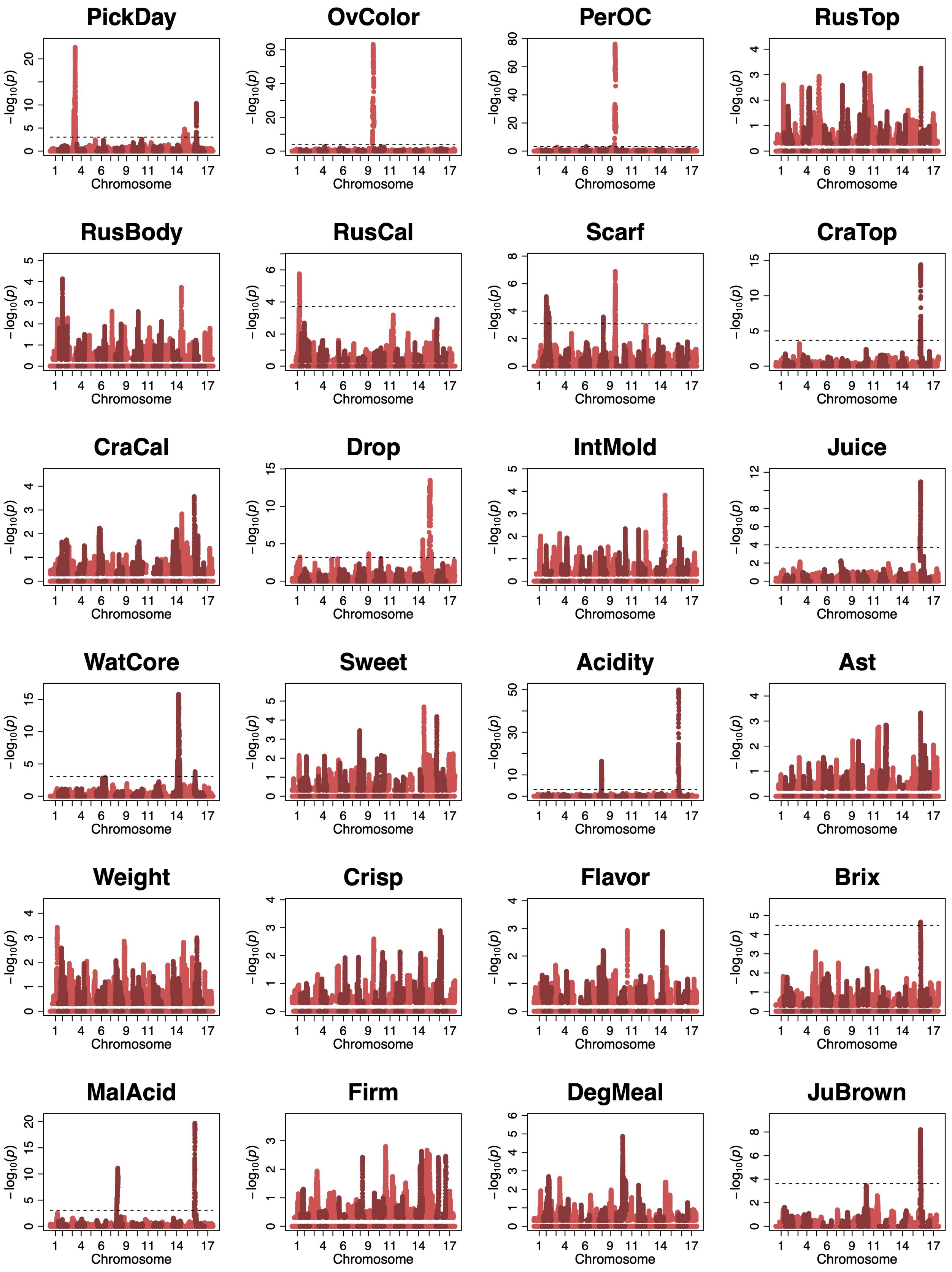

Supplementary Figure S3. SNP-set GWAS using combined GRAS-Di and Infinium marker dataset.
The combined GRAS-Di and Infinium marker dataset is shown in Fig. 2A. Dashed lines indicate a false discovery rate of 0.05.

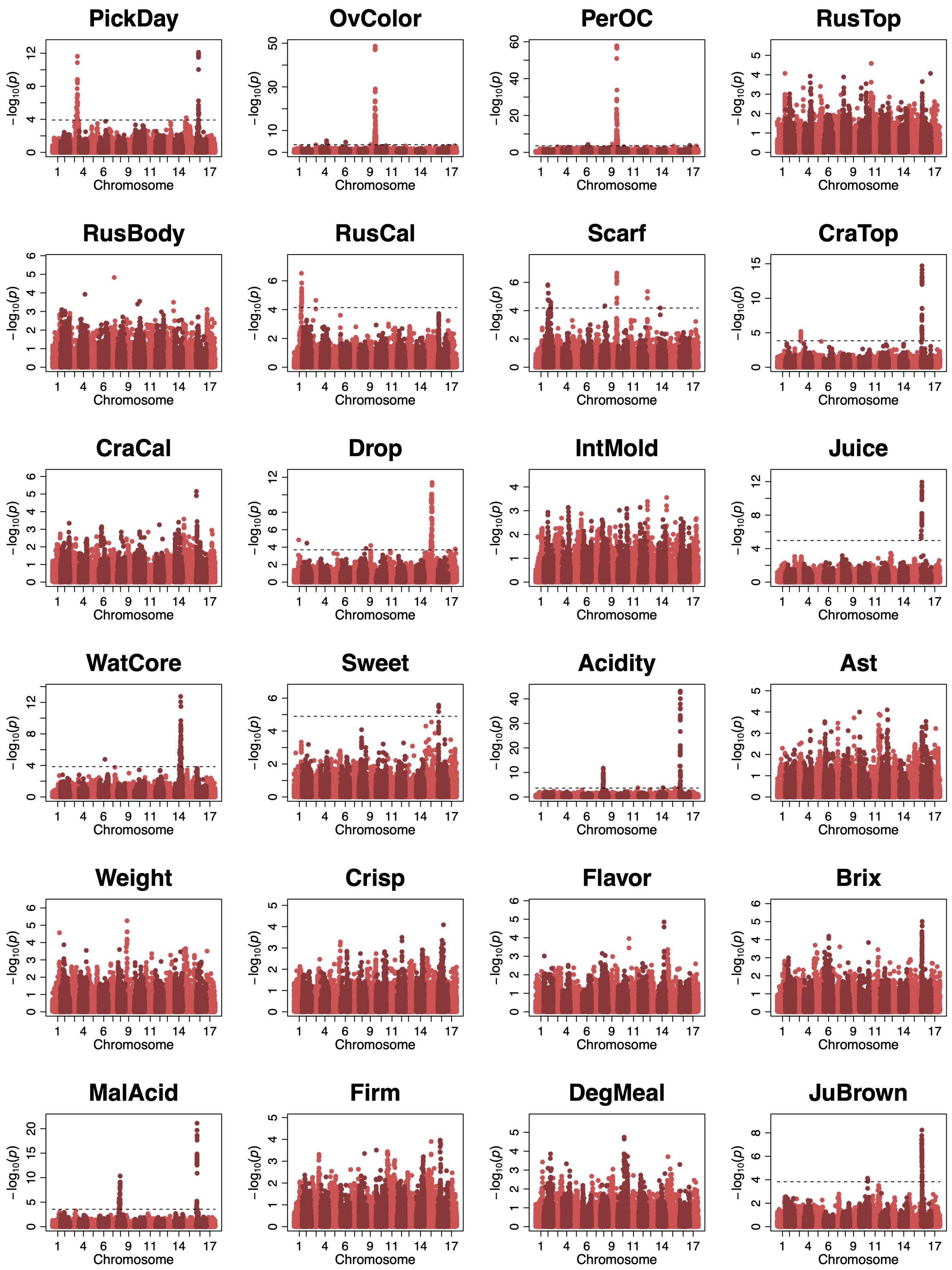

Supplementary Figure S4. Single-SNP GWAS using combined GRAS-Di and Infinium marker dataset.

The combined GRAS-Di and Infinium marker dataset is shown in Fig. 2A. Dashed lines indicate a false discovery rate of 0.05.

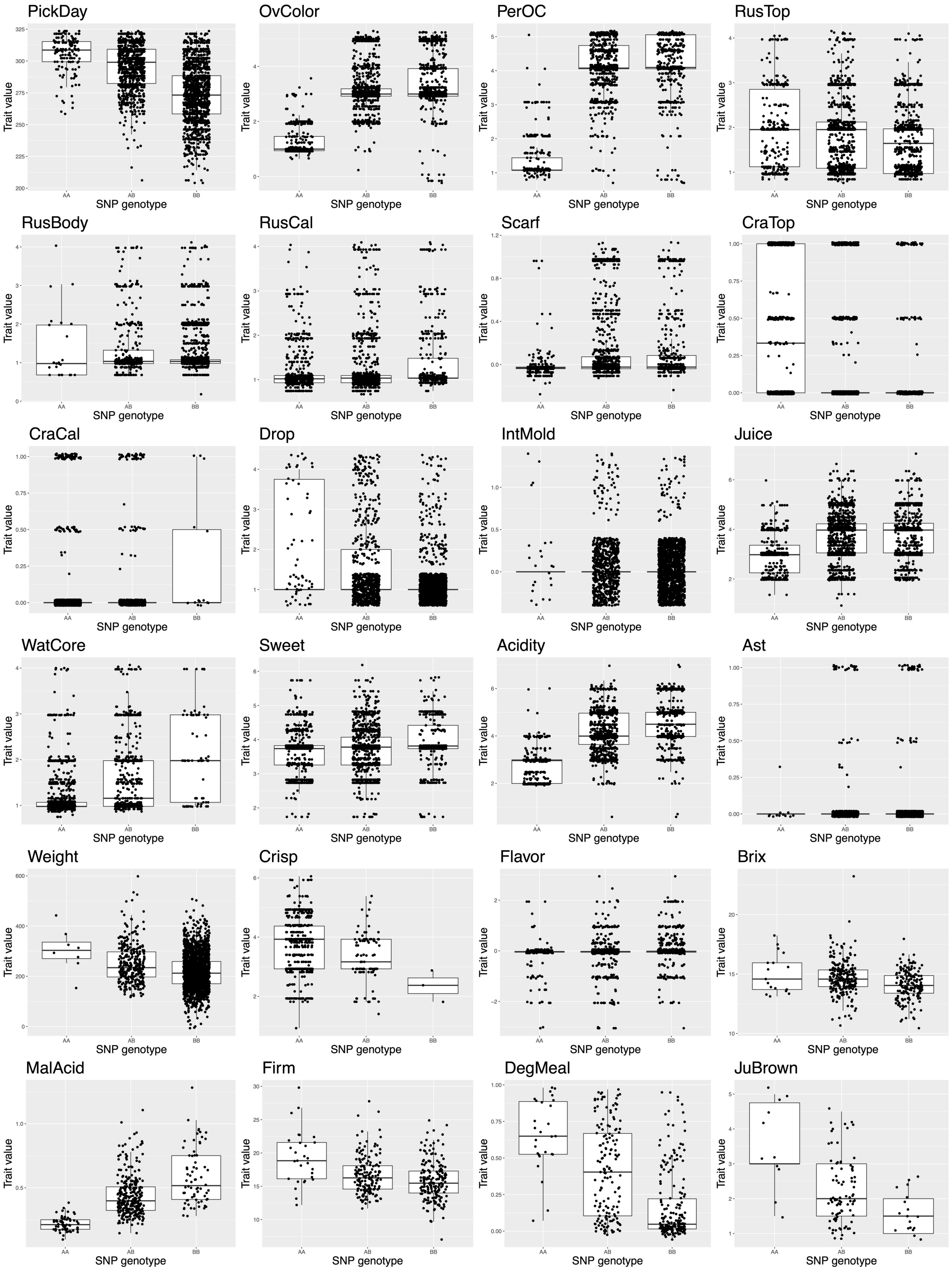

Supplementary Figure S5. Associations between the top peak SNP genotypes in single-SNP GWAS using combined data and trait phenotypes.
The combined GRAS-Di and Infinium marker dataset is shown in Fig. 2A.

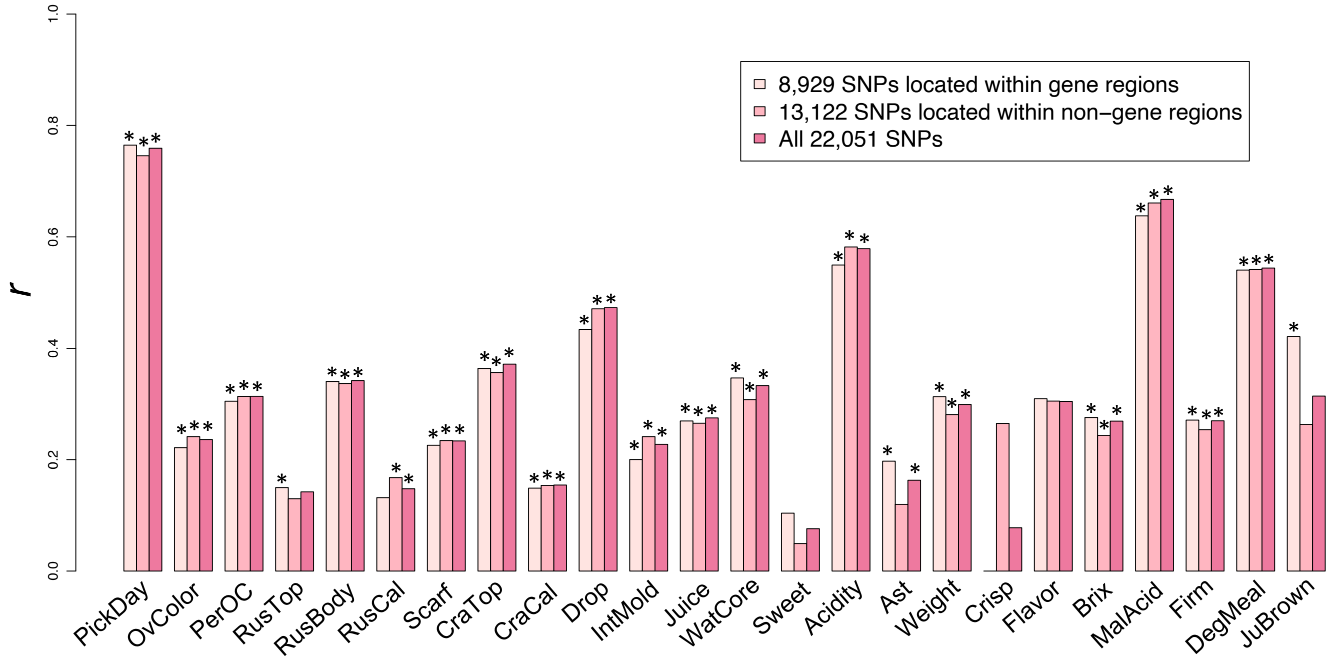

Supplementary Figure S6. Effect of different number of SNPs on prediction accuracy. Accuracy of genomic prediction for 24 fruit traits was evaluated using the Pearson's correlation coefficient (*r*) between the predicted genotypic values and phenotypic values. When the estimated *r* was below zero, it was regarded as zero.

Asterisks indicate statistically significant correlations:*$p < 0.05$.

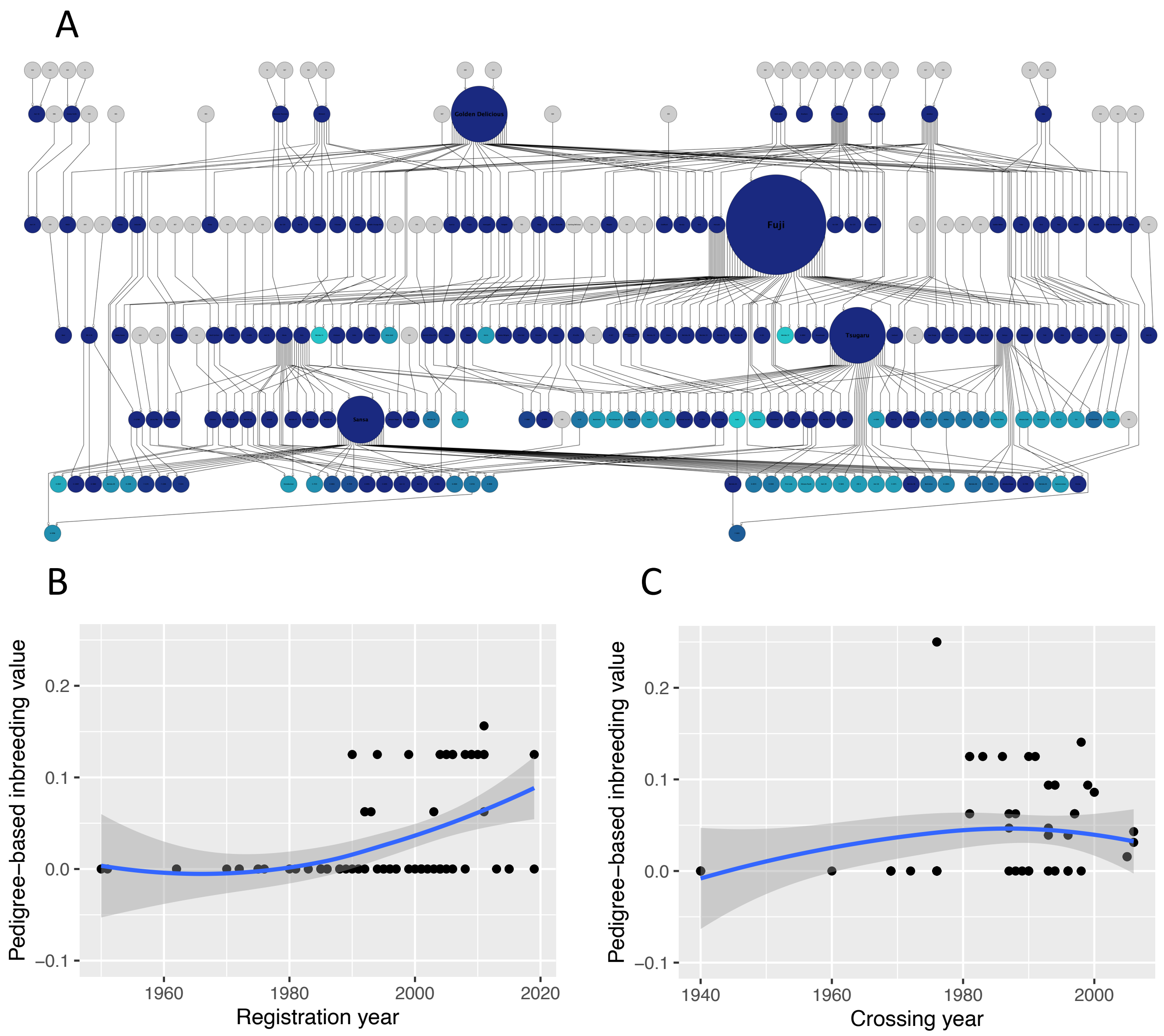

Supplementary Figure S7. Changes in pedigree-based inbreeding coefficients in apple parental population

(A) Visualization of the pedigree-based inbreeding coefficients on the pedigree of the apple parental varieties. The blue circles indicates the varieties used in this study. Lighter or darker blue circles show higher or lower values, respectively. The size of the circles is based on pedigree contribution. Relation between the marker genotype-based inbreeding coefficients and registration (B) or crossing (C) years of apple parental varieties. Blue lines indicate local polynomial regression fitting with 0.95 confidence intervals indicated in dark grey.

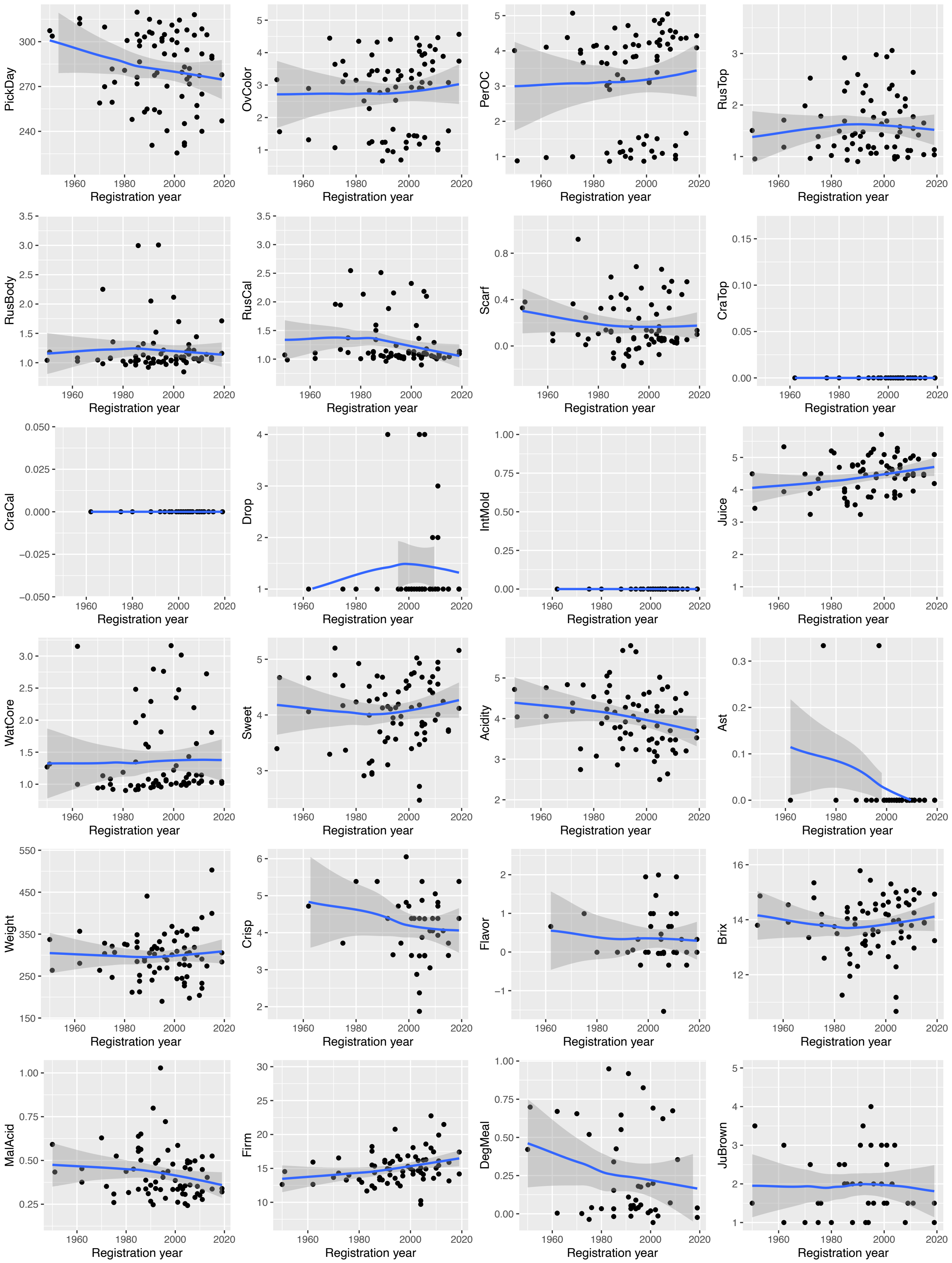

Supplementary Figure S8. Relationship between phenotypic values of fruit traits and registration years in parental population.

Blue lines indicate regression lines with 0.95 confidence intervals indicated in dark greys.

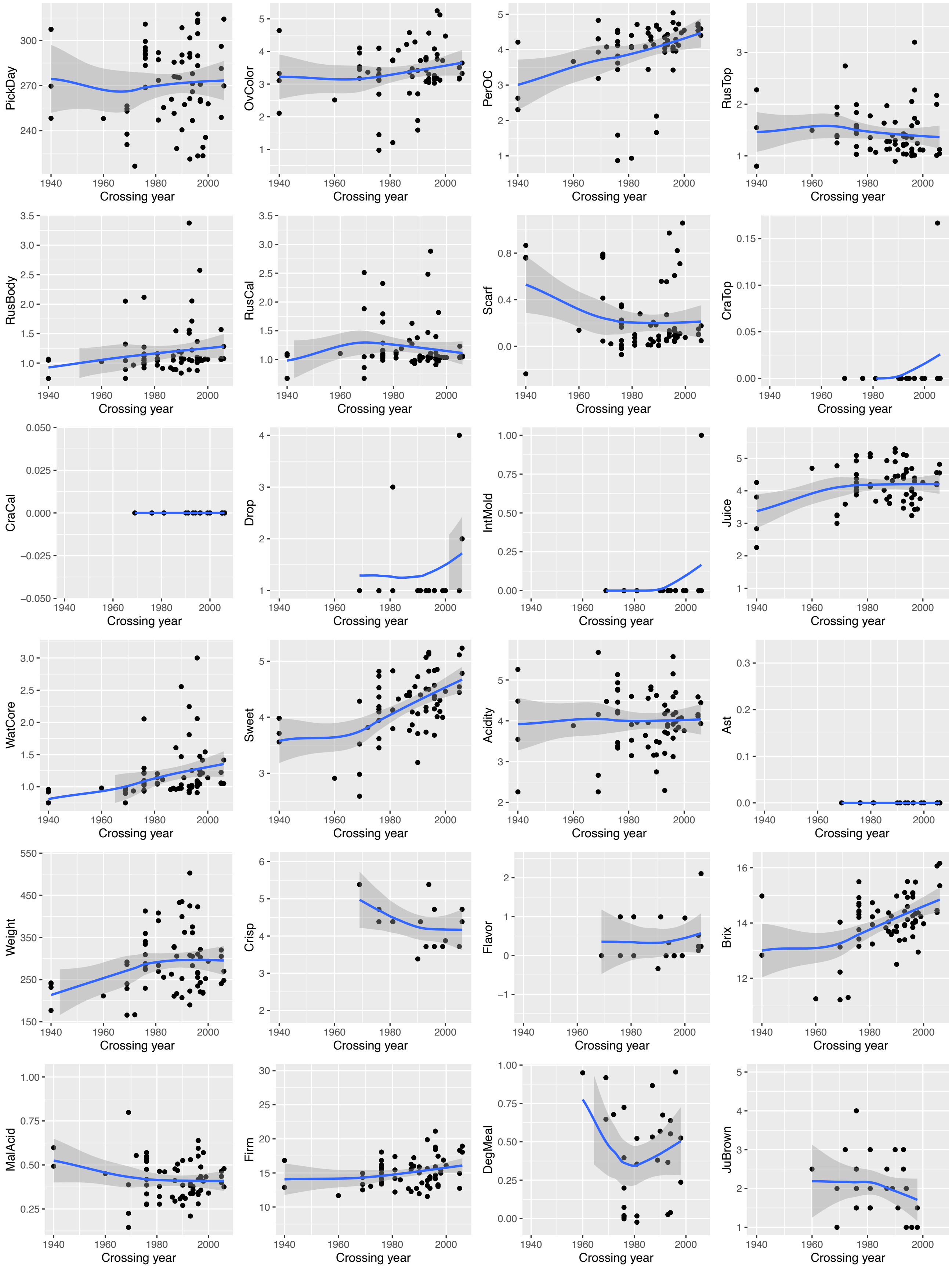

Supplementary Figure S9. Relationship between phenotypic values of fruit traits and crossing years in parental population.

Blue lines indicate regression lines with 0.95 confidence intervals indicated in dark greys.
